# Identification of functionally distinct and interacting cancer cell subpopulations from glioblastoma with intratumoral genetic heterogeneity

**DOI:** 10.1093/noajnl/vdaa061

**Published:** 2020-05-27

**Authors:** Min Guo, Marjolein van Vliet, Jian Zhao, Teresita Díaz de Ståhl, Mikael S Lindström, Huaitao Cheng, Susanne Heller, Monica Nistér, Daniel Hägerstrand

**Affiliations:** 1 Department of Oncology-Pathology, Karolinska Institutet, Stockholm, Sweden; 2 Department of Medical Biochemistry and Biophysics, Karolinska Institutet, Stockholm, Sweden; 3 Uppsala Clinical Research Center (UCR), Uppsala University, Uppsala University Hospital, Uppsala, Sweden

**Keywords:** cell-to-cell interaction, glioblastoma heterogeneity, NOTCH, secretome, temozolomide, TGFBI

## Abstract

**Background:**

Glioblastomas display a high level of intratumoral heterogeneity with regard to both genetic and histological features. Within single tumors, subclones have been shown to communicate with each other to affect overall tumor growth. The aim of this study was to broaden the understanding of interclonal communication in glioblastoma.

**Methods:**

We have used the U-343 model, consisting of U-343 MG, U-343 MGa, U-343 MGa 31L, and U-343 MGa Cl2:6, a set of distinct glioblastoma cell lines that have been derived from the same tumor. We characterized these with regard to temozolomide sensitivity, protein secretome, gene expression, DNA copy number, and cancer cell phenotypic traits. Furthermore, we performed coculture and conditioned media-based experiments to model cell-to-cell signaling in a setting of intratumoral heterogeneity.

**Results:**

Temozolomide treatment of a coculture composed of all 4 U-343 cell lines presents a tumor relapse model where the least sensitive population, U-343 MGa 31L, outlives the others. Interestingly, the U-343 cell lines were shown to have distinct gene expression signatures and phenotypes although they were derived from a single tumor. The DNA copy number analysis revealed both common and unique alterations, indicating the evolutionary relationship between the cells. Moreover, these cells were found to communicate and affect each other’s proliferation, both via contact-dependent and -independent interactions, where NOTCH1, TGFBI, and ADAMTS1 signaling effects were involved, respectively.

**Conclusions:**

These results provide insight into how complex the signaling events may prove to be in a setting of intratumoral heterogeneity in glioblastoma and provide a map for future studies.

**Key Points**

- An intratumoral heterogeneous model, U-343, is used.
- Chemotherapy-resistant clones exist in the tumors. They will grow up and cause tumor relapse after treatment.
- There are interactions between heterogeneous clones, which are potential therapeutic targets.

**Importance of the Study**

This study shows that genetically distinct cell populations from a single glioblastoma tumor have different drug sensitivities and phenotypes related to astrocytic versus mesenchymal features. Also, the glioblastoma subtype phenotype was affected in a coculture setting. Furthermore, NOTCH, TGFBI, and ADAMTS1 signaling are indicated as modulators of intratumoral communication in glioblastomas. In summary, we show that the U-343 cell line panel serves as a useful model to study intratumoral heterogeneity.

Glioblastoma is the most aggressive brain tumor in adults with a median survival of 15 months.^1^ Current treatment consists of surgery followed by radiotherapy and adjuvant temozolomide. Single glioblastomas have been shown to consist of an intermixture of cancer cell populations representing the previously described subclasses: classical, proneural, and mesenchymal.^2–4^ Recent findings in several cancer types support the view that tumors may contain several subpopulations of cells with different genotypic and phenotypic properties, including different response to therapy.^5–10^

Within the same glioblastoma, different cancer cell populations have been described with distinct genetic alterations including amplification of platelet-derived growth factor receptor alpha (*PDGFRA*) in combination with either epidermal growth factor receptor (*EGFR*) or *MET* (hepatocyte growth factor receptor) amplification.^11,12^ Furthermore, multiple studies have shown that intratumoral genetic heterogeneity is frequently occurring in glioblastoma, where different cancer cell subpopulations may communicate and depend on each other, like in a social network.^13,14^

To study the effect of heterogeneity on overall tumor cell interactions, we have used a glioma model that consists of a panel of cell lines derived from one single glioblastoma.^15,16^ Here we have analyzed how these cancer cell lines act during chemotherapy, how they phenotypically and genotypically differ, and how they communicate via direct cell-to-cell contact and secreted factors.

## Materials and Methods

Only basic information is provided in this section. More detailed information can be found in the supplementary material.

### Cell Culture Conditions

**Figure 1.**
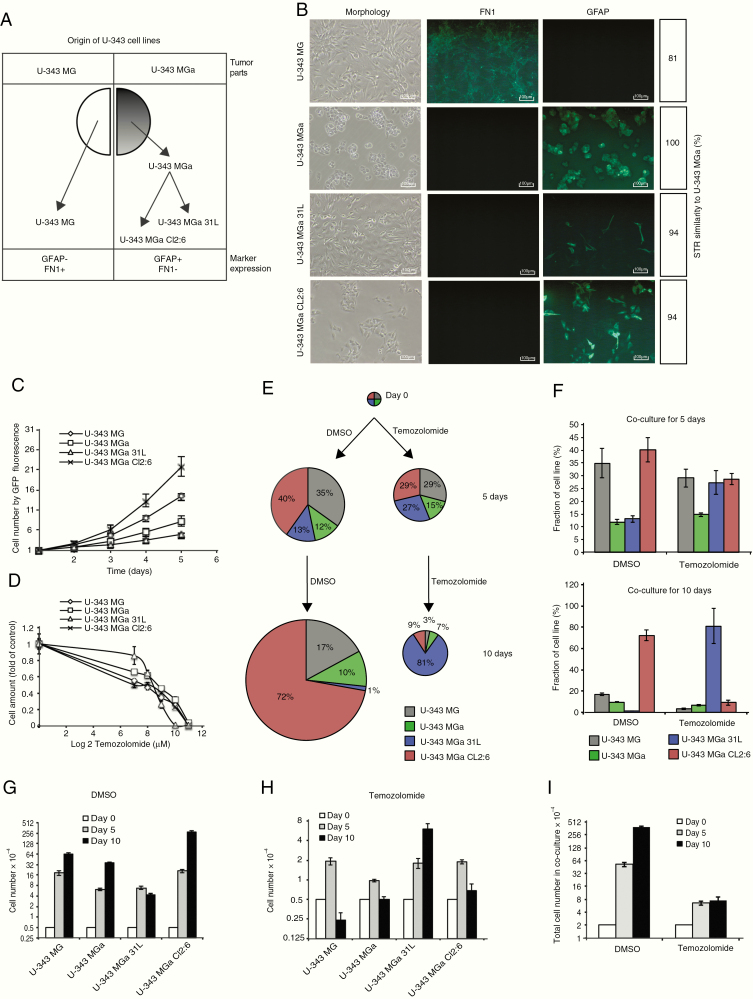
Coculture of all 4 U-343 cell lines mimics the behavior of drug-resistant tumor cell clones upon temozolomide treatment. (A) The model for origin of U-343 MG, U-343 MGa, U-343 MGa 31L, and U-343 MGa Cl2:6, all derived from a single glioblastoma tumor by subcloning and maintained as cell lines. (B) Individual U-343 cell lines morphology, GFAP and FN1 immunofluorescence staining, and the 3 other cell lines similarity with U-343 MGa monitored by STR. (C) Growth curve of GFP-labeled U-343 cell lines measured by GFP fluorescence. (D) Temozolomide sensitivity profiles of U343 cell lines measured by MTT assay. About 3500 cells were seeded in 96-well plates and treated with temozolomide (concentration range from 0 to 2000 µM) for 4 days. (E) Assessment of population balances during coculturing of all 4 U-343 cell lines in the presence and absence of temozolomide. (F) Percentage of each cell line after coculturing for 5 (upper panel) and 10 days (lower panel) in the presence of dimethyl sulfoxide (DMSO) or 200 µM temozolomide. (G and H) Individual cell line numbers after coculturing for 5 and 10 days in the presence of DMSO (G) or 200 µM temozolomide (H). (I) Total U-343 cell number in the coculture after 5 and 10 days in the presence of DMSO or 200 µ M temozolomide.

The high-grade human glioma cultures, the U-343 cell panel, including U-343 MG, U-343 MGa, U-343 MGa 31L, and U-343 MGa Cl2:6, were retrieved from a local cell culture bank (Department of Immunology, Genetics and Pathology, Uppsala University, Sweden) and cultured as previously described.^15–17^ U-343 MG cells express fibronectin 1 (FN1) but not glial fibrillary acidic protein (GFAP), and conversely the U-343 MGa cultures express GFAP but not FN1 (Figure 1A and B).

### Immunofluorescence Staining, Western Blotting, and Real-Time PCR

Immunofluorescence, western blotting, and real-time PCR were performed as previously described.^18^ Antibodies and primers are provided in Supplementary Table S1.

### RNA-seq and Genetic Analysis

RNA and genomic DNA were isolated from the U-343 cells. RNA was used for RNA-sequencing (RNA-seq). RNA-seq data have been deposited at the EBI ArrayExpress database (accession number E-MTAB-8620). DNA was used for somatic copy number analysis.

### Generation of GFP Labeled, Knockdown, and NOTCH1 Knockout Cells

Green Florescent Protein (GFP)-expressing cells and gene suppression cells by short hairpin ribonucleic acid (shRNA) were generated by lentiviral transduction as previously described.^19^*NOTCH1* knockout was performed by CRISPR/Cas9 based gene editing.^20^

### Cell Invasion and Proliferation Assays

The invasion capacity of U-343 cells was measured by the Matrigel invasion assay. Cell proliferation was assessed by fluorescence measurements with Tecan microplate reader (Tecan) or by MTT assay.

### Temozolomide Treatment

Temozolomide sensitivity in U-343 cell lines was tested by the addition of temozolomide at different concentrations and assessed by MTT assay. In the coculture experiments, the 4 U-343 cell lines were mixed (25% of each) and cultured in the presence of Dimethyl sulfoxide (DMSO) or 200 µM temozolomide for 5 and 10 days.

### Conditioned Media and Cell-to-Cell Coculture Experiments

Conditioned media was added in a 1:1 ratio mix with unconditioned media to U-343 cell lines and cultured for 7 days. In coculture experiments, non-GFP-labeled cells were seeded on the bottom for 48 h, followed by addition of GFP-labeled cells on top, and cultured for 5 days.

### Secretome Analysis by Click-iT

To detect secreted proteins we used Click-iT Protein Enrichment Kit according to a previously described Click-iT protocol.^21^ Cells were incubated with methionine-free DMEM supplemented with either methionine or L-azidohomoalanine (methionine analog with reactive azide group). Collected conditioned media was concentrated and secreted proteins were bound to Click-iT beads by click chemistry. Bound proteins were subsequently digested and identified by mass spectrometry.

### Statistical Analysis

Bar graphs generated in Excel with error bars show average ± standard deviation. The identification of significant differences in average between groups was performed by Student’s *t*-test and the correlation between parameters was assessed by Pearson’s tests.

## Results

### The U-343 Cell Panel Provides a Valuable Model to Study the Role of Intratumoral Heterogeneity in Temozolomide Resistance

The U-343 cell panel, composed of U-343 MG, U-343 MGa, U-343 MGa 31L, and U-343 MGa Cl2:6, has previously been described (Figure 1A).^15–17^ Here, the identity of the cell lines was confirmed by immunofluorescence staining showing expression of FN1 in U-343 MG and GFAP in the 3 U-343 MGa cultures and by short tandem repeat (STR) analysis (Figure 1B; Supplementary Materials and Methods). Proliferation rates were determined where the highest was found for U-343 MGa Cl2:6 followed by U-343 MG, U-343 MGa, and U-343 MGa 31L, in decreasing order (Figure 1C). To assess differences in sensitivity to temozolomide, we conducted drug titration experiments and found U-343 MGa 31L was the least sensitive cell line compared with the other 3 cell lines in different conditions, such as short incubation time (Figure 1D, 3500 cells treated for 4 days), longer incubation time (Supplementary Figure S1A, 3500 cells treated for 8 days), or higher cell amount (Supplementary Figure S1B, 10 000 cells treated for 4 days). Subsequently, all the 4 U-343 cell lines were cocultured in the presence or absence of temozolomide to investigate cooperative effects on temozolomide sensitivity, overall tumor growth, and population balance. After 10 days of coculture, U-343 MGa Cl2:6 became the most abundant clone representing 72% of the coculture population, followed by U-343 MG, U-343 MGa, and U-343 MGa 31L at 17%, 10%, and 1%, respectively (Figure 1E–H). On the contrary, in the presence of 200 µM temozolomide, U-343 MGa 31L became the most prevalent clone at 81%, followed by U-343 MGa Cl2:6, U-343 MGa, and U-343 MG at 9%, 7%, and 3%, respectively (Figure 1E–H). After 10 days of growth with temozolomide, the total amount of cells was 50-fold less as compared to the untreated culture, indicating a general decrease in cell growth ( 75 000 vs 3 840 000 cells) (Figure 1I). Still, the absolute cell number of U-343 MGa 31L increased during temozolomide treatment as opposed to the other cell lines (Figure 1G and H). In conclusion, U-343 MGa 31L became the least represented clone in coculture without temozolomide, whereas it became the most represented clone during temozolomide treatment. As a temozolomide-resistant clone, U-343 MGa 31L thus outgrew the others under drug selection pressure and became the dominating clone. This shows the U-343 cell panel as an in vitro model for tumor relapse in a setting of intratumoral heterogeneity.

### The U-343 Cell Lines Display Phenotypic Differences in Gene Expression and Cell Motility

**Figure 2.**
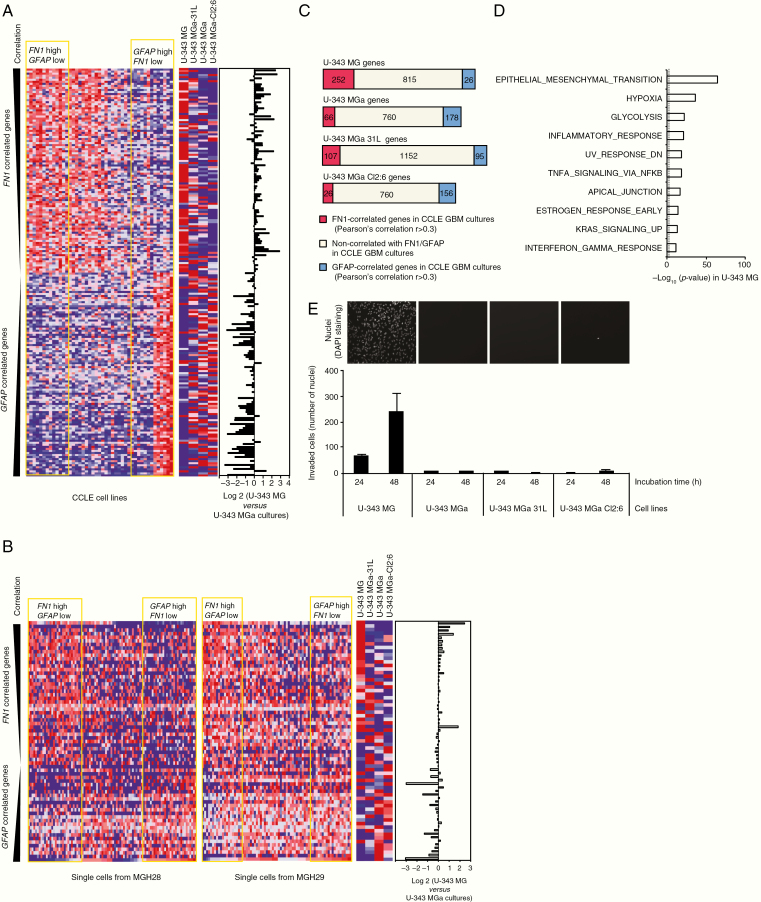
Glioblastoma U-343 cell lines display phenotypic and gene expression differences. (A) Heatmap showing expression of the top 100 highly *FN1*-correlated and *GFAP*-correlated genes in *FN1*^high^/*GFAP*^low^ and *GFAP*^high^/*FN1*^low^ cell lines from CCLE database (left panel). The right panel displays the expression of these genes in U-343 cell lines based on RNA-seq data (see Supplementary Table S3). The bar graph in the right shows the ratio of gene expression in U-343 MG versus average of U-343 MGa cultures. (B) Same as in (A), but for the analysis of single-cell RNA-sequencing data from 2 single glioblastomas MGH28 and MGH29 (see Supplementary Table S4). (C) Strip charts displaying the number of genes highly expressed in each U-343 cell line compared to the average of others. Genes correlated with *FN1* (red, Pearson’s test *r* > 0.3) and *GFAP* (blue, *r* > 0.3) expression in CCLE cell lines were used in the analysis. (D) Hallmark gene categories processed by GenePattern among U-343 MG genes versus an average of U-343 MGa subclones (>2 fold). (E) Matrigel invasion assay performed with U-343 cell lines. Invaded cells were calculated by nuclear counting as shown by representative images above graph at 48 hours’ incubation.

To investigate cancer-related phenotypes of the U-343 cell lines, we performed RNA-seq-based gene set enrichment analysis (GSEA), glioblastoma subset analysis, and Matrigel invasion assays. We identified genes higher expressed in each cell line compared to the average expression in the other 3 cell lines (>2-fold). These genes were denominated: “U-343 MG genes” (1093 genes), “U-343 MGa genes” (1004 genes), “U-343 MGa 31L genes” (1354 genes), and “U-343 MGa Cl2:6 genes” (942 genes) (Supplementary Table S2). To analyze the U-343 gene expression signatures in the context of other glioblastoma cell lines, we compared these with data from 45 glioblastoma cell lines retrieved from the Cancer Cell Line Encyclopedia (CCLE). Since U-343 MG exclusively expressed *FN1*, and the other U-343 cell lines expressed *GFAP*, the CCLE cell lines were divided into 4 groups according to the median value of *FN1* and *GFAP*. The gene expression signature of U-343 MG matched *FN1*^*high*^*GFAP*^*low*^ expressing CCLE cell lines, whereas the signatures of U-343 MGa cell lines resembled the signature of *GFAP*^*high*^*FN1*^*low*^ expressing CCLE cell lines (Figure 2A; Supplementary Table S3). To further investigate the generalizability of different states within individual glioblastomas, we analyzed single-cell RNA-seq data from glioblastomas MGH28 and MGH29.^2^ Similar as in CCLE cell lines, we identified *FN1*^*high*^*GFAP*^*low*^ and *GFAP*^*high*^*FN1*^*low*^ expressing cell groups, which also matched with U-343 signature (Figure 2B; Supplementary Table S4). Furthermore, we found that 23% (252/1093) of *FN1-*correlated genes in CCLE cell lines (Pearson’s correlation *r* >0.3) overlapped with “U-343 MG genes” and conversely, 18% (178/1004), 7% (95/1354), and 17% (156/942) of *GFAP-*correlated genes (*r* > 0.3) overlapped with “U-343 MGa genes,” “U-343 MGa 31L genes,” and “U-343 MGa Cl2:6 genes,” respectively (Figure 2C). A GSEA of hallmark gene sets for highly expressed genes in each corresponding cell line identified enrichment for several gene sets (Figure 2D; Supplementary Figure S2A–D and Supplementary Table S5). Of particular note, the epithelial to mesenchymal transition gene set was the top hit for “U-343 MG genes” (77 genes) and at 11th, 6th, and 11th positions for “U-343 MGa genes” (17 genes), “U-343 MGa 31L genes” (25 genes), and “U-343 MGa Cl2:6 genes” (21 genes), respectively (Figure 2D; Supplementary Figure S1A–D). At a closer look the epithelial to mesenchymal transition gene set genes did not overlap (Supplementary Figure S2E and F). The genes enriched in U-343 MG included classical mesenchymal genes *FN1*, *SNAI2*, and *COL1A1*. Furthermore, U-343 MG had a high support index for mesenchymal glioblastoma subtype, calculated as previously described,^3,18^ compared to the U-343 MGa cell lines (Supplementary Figure S2G). Thus, the U-343 MG mesenchymal signature represents that of a glioblastoma mesenchymal subtype gene expression signature. To extend this finding, we performed Matrigel invasion assay and found that U-343 MG had a higher in vitro Matrigel invasion capacity as compared to the other U-343 cell lines (Figure 2E).

In summary, the patterns of *FN1* and *GFAP* correlated genes, a glioblastoma mesenchymal subtype signature, and the Matrigel invasion capacity indicate that U-343 MG has a more mesenchymal phenotype compared to the U-343 MGa cultures. Overall, these findings indicate that cultured cells from a single glioblastoma tumor may display different gene expression signatures and connected phenotypic traits.

### The U-343 Cell Lines Share a Common Tumor Cell Ancestor Based on DNA Copy Number Analysis

**Figure 3.**
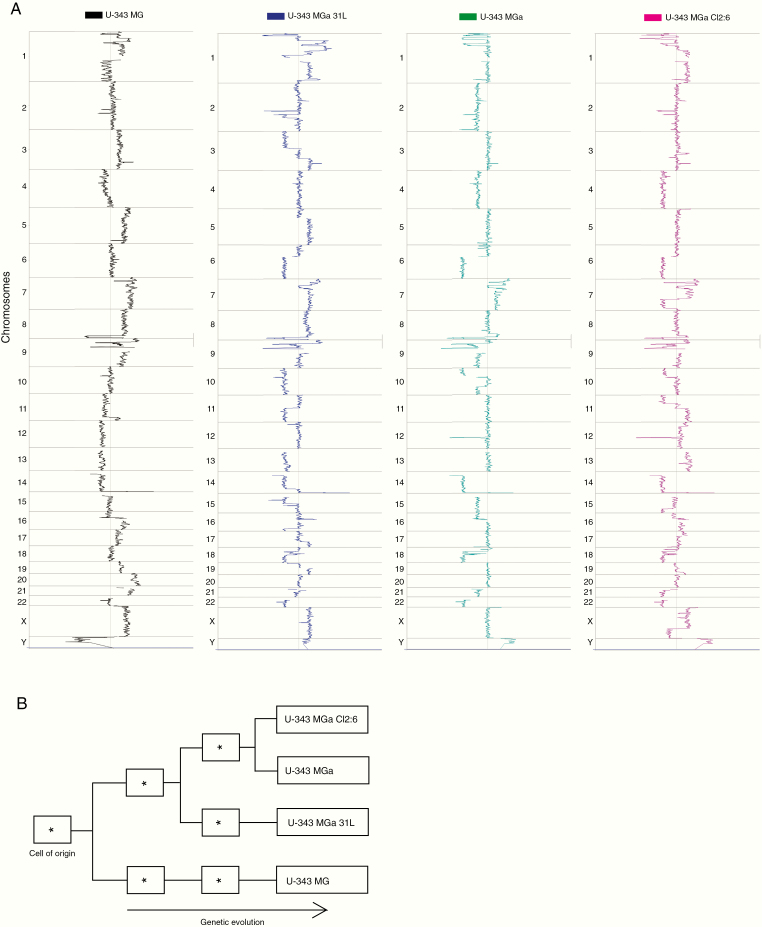
Copy number analyses of U-343 cell lines reveal common and specific genomic alterations and clonal evolution. (A) The overall spectrum of chromosomal copy number alterations in the U-343 cell lines. The curve on the right side of each central line indicates the gain of chromosome (DNA) and to the left loss. (B) Clonal relationships based on copy number alterations.

To determine the genetic relationship between the different U-343 cell lines, we performed DNA copy number analysis (Figure 3A) by comparing whole chromosomal, partial, and focal alterations. The U-343 cell lines had in common gain of chromosome 7 and loss of chromosome 14q, which harbor *EGFR* and *CDK6*, and *AKT1*, respectively.^22^ Furthermore, they shared focal gains at 3q26.1, 4q13.3, and 16p11.2 and focal losses at 1p32.2, 1p36.22, 2q24.3, 7p21.1, 8q24.23, 9p21.3, 10q11.22, and 22q12.1. The U-343 MGa cell lines shared loss of 6q, 10p, 18q, and 22q. The U-343 MGa cultures also shared focal gain at 18q12.1 and loss at 7p14.1. Beyond these shared alterations, the U-343 cell lines displayed multiple unique alterations (Supplementary Figure S3A). Cell line-specific altered genes were compared with gene expression level from the RNA-seq data to identify genes whose gene expression level may be affected due to DNA copy level alterations (Supplementary Figure S3B and Supplementary Table S6). Interestingly, *CDKN1B* (p27, Kip1), a known regulator of cell proliferation often lost in glioblastoma,^23^ was found to have a lower gene copy number and gene expression in U-343 MGa 31L.

By hierarchical clustering, the clonal relationship was investigated based on copy number alterations (Figure 3B). The pattern of shared and specific alterations suggested a relationship where U-343 MG diverged from the other clones at an early stage. Subsequently, the U-343 MGa clones are suggested to be more closely related in tumor evolution, where U-343 MGa-31L diverged early from U-343 MGa and U-343 MGa Cl2:6. In summary, U-343 MG is the most divergent cell line, whereas the U-343 MGa cell lines constitute a group in which U-343 MGa-31L seems to have diverged early from U-343 MGa and U-343 MGa Cl2:6.

### NOTCH1 Mediates a Proliferation Inhibitory Effect of U-343 MG on U-343 MGa Cl2:6

**Figure 4.**
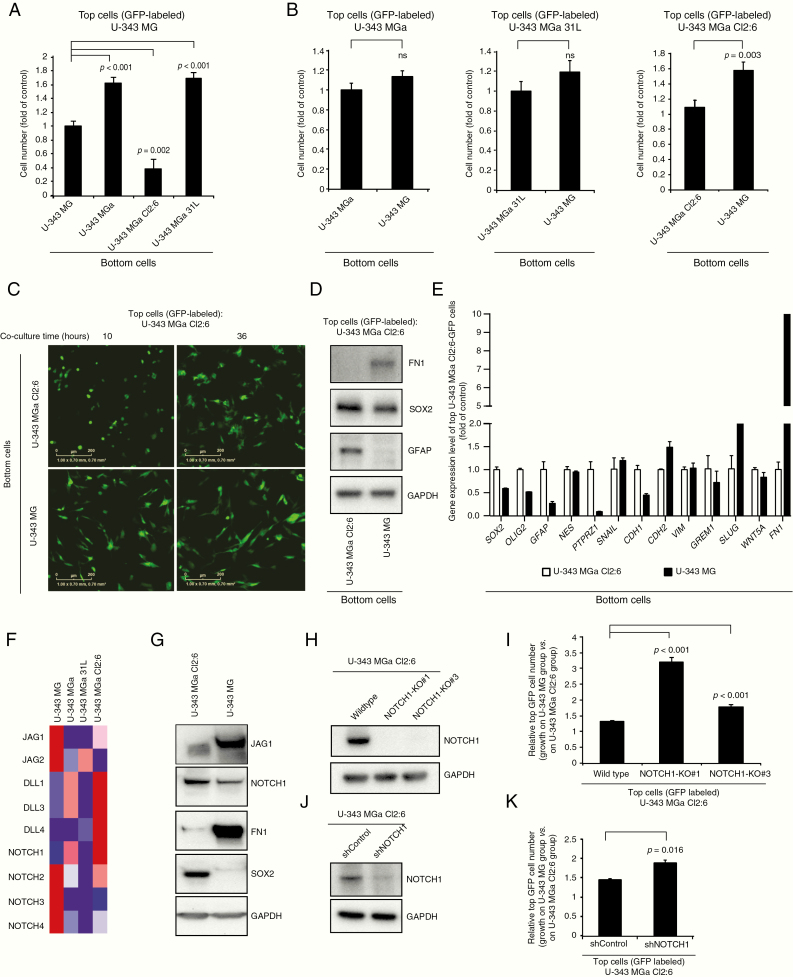
Cell-to-cell contacts in glioblastoma cell cultures uniquely affect the proliferation of individual lines, with NOTCH1 signaling playing a significant role in astrocytic cells. (A) A relative number of GFP-labeled U-343 MG cells after culturing for 5 days on top of non-labeled confluent U-343 cell lines. (B) Relative cell number of GFP-labeled U-343 MGa, U-343 MGa 31L, and U-343 MGa Cl2:6 cells after culturing for 5 days on top of confluent U-343MG or themselves as a control. (C) The real-time images of GFP-labeled U-343 MGa Cl2:6 cells after coculture on top of U-343 MGa Cl2:6 (top panel) or U-343 MG (lower panel) for 10 h (left) or 36 h (right). (D and E) Differential levels of FN1, SOX2, and GFAP proteins by Western blot (D) or relative gene expression of selected stemness/neural and mesenchymal markers (E) in sorted GFP-labeled U-343 MGa Cl2:6 cells after coculture with U-343 MGa Cl2:6 (empty bar) or U-343 MG (filled bar). (F) Relative gene expression pattern of NOTCH receptors and ligands in U-343 cell lines by RNA-seq data. Red and blue colors indicate relatively high and low expression levels, respectively. (G) Western blot showing protein levels of JAG1, NOTCH1, FN1, and SOX2 in U-343 MGa Cl2:6 and U-343 MG. (H) Western blot showing NOTCH1 protein level in NOTCH1-KO#1 and NOTCH1-KO#3 CRISPR/Cas9-generated U-343 MGa Cl2:6 clones. (I) Relative cell number of GFP-labeled U-343 MGa Cl2:6, NOTCH1-KO#1, and NOTCH1-KO#3 after 5 days of culture on top of confluent wild-type U-343 MGa Cl2:6 and U-343MG. (J) Western blot showing NOTCH1 protein level in shRNA control and shNOTCH1 U-343 MGa Cl2:6. (K) Same as (I), but for shRNA control and shNOTCH1 U-343 MGa Cl2:6 cells. ns, non-significant.

To identify interclonal effects on cell proliferation, we performed coculture experiments with mixed pairs (seeded in a ratio of 1 top: 5 bottom cells) (Figure 4A and B). The number of U-343 MG cells increased by 62% and 69% when grown on top of U-343 MGa and U-343 MGa 31L, respectively, and decreased by 62% when grown on top of U-343 MGa Cl2:6 (Figure 4A). Conversely, U-343 MGa Cl2:6 cells increased by 44% when grown on top of U-343 MG (Figure 4B, right panel), whereas U-343 MGa or U-343 MGa 31L cells were not significantly affected (Figure 4B, left and middle panels). Furthermore, we observed the morphology of U-343 MGa Cl2:6 cells became elongated when grown on top of U-343 MG (Figure 4C). To further investigate this morphological change, U-343 MGa Cl2:6 (GFP) cells were subsequently FACS-sorted after coculture with U-343 MG or with U-343 MGa Cl2:6 as control. Western blot analyses identified increased FN1 and decreased GFAP and SOX2 protein levels (Figure 4D). Real-time PCR analysis suggested that U-343 MGa Cl2:6 became more mesenchymal and less progenitor-like based on increased expression of mesenchymal genes including *CDH2* (N-cadherin), *SNAI2* (SLUG), and *FN1* and decreased expression levels of markers for neural stem and progenitor cells including *SOX2*, *OLIG2*, *GFAP*, *PTPRZ1*, and *CDH1* (E-cadherin) (Figure 4E). In summary, these results show that heterogeneous tumor cells from a glioblastoma can affect each other by cell-to-cell interactions, affecting cell proliferation rate, cell morphology, and gene expression patterns. In particular, the mesenchymal-like U-343 MG cells transitioned the non-mesenchymal U-343 MGa Cl2:6 cells to a mesenchymal-like phenotype paralleled by an increased growth rate in vitro.

To elucidate the mechanisms behind the cell-to-cell mediated growth promotion of U-343 MGa Cl2:6 by U-343 MG, we examined gene and protein expression levels of NOTCH-signaling components. NOTCH signaling has been shown to influence cell proliferation, survival, and apoptosis and also to play an important role during brain development and glial cell differentiation through direct cell-to-cell contact.^24^ We thus hypothesized that NOTCH signaling may be involved in cell-to-cell communication between U-343 MG and U-343 MGa Cl2:6. Specifically, *JAG1* and *JAG2* were highly expressed in U-343 MG, whereas *NOTCH1* was highly expressed in U-343 MGa Cl2:6 (Figure 4F and G). By *NOTCH1* knockout in U-343 MGa Cl2:6 2 clones were generated denoted as NOTCH1-KO #1 and KO #3. The knockout effect was confirmed by Sanger sequencing (Supplementary Figure S4A and B) and Western blot (Figure 4H), and the cells were GFP labeled for coculture experiments. The growth rates of *NOTCH1* WT, KO #1, and #3 U-343 MGa Cl2:6 cells were significantly increased by 1.32, 3.14, and 1.78-fold when cultured on top of U-343 MG, as compared to on top of nonmodified U-343 MGa Cl2:6 cells (Figure 4I). We recapitulated these findings by shRNA suppression of *NOTCH1* in the same cells (Figure 4J and K). We thus conclude that NOTCH1 signaling in U-343 MGa Cl2:6 normally exerts an inhibitory effect on (protection from) unknown growth-promoting stimuli mediated by U-343 MG when in direct cell-to-cell contact.

### U-343 Cell Lines Mutually Affect Each Other’s Proliferation via Secreted Proteins

**Figure 5.**
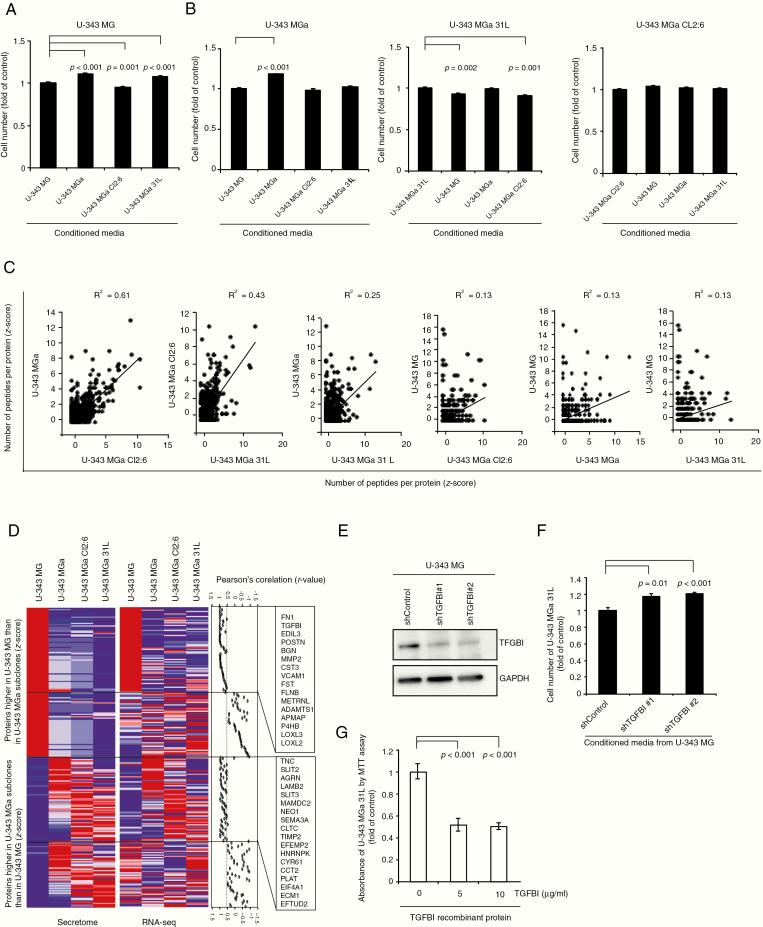
U-343 heterogeneous cell lines affect each other’s proliferation by secreted proteins. (A) Relative cell number of U-343 MG after culturing for 7 days in the different conditioned media from all U-343 cell lines as indicated. Cells cultured in own conditioned media were used as control. (B) Relative cell number of U-343 MGa, U-343 MGa 31L, and U-343 MGa Cl2:6 after culturing for 7 days in conditioned media from all U-343 cell lines. (C) Comparison of relative amounts of secreted proteins identified by mass spectrometry in conditioned media from different U-343 cell lines. The sample comparisons are arranged from left to right by order of similarity as determined by Pearson’s correlation (*R*^2^ values). (D) Identification of 4 groups of secreted proteins based on the correlation between protein and gene expression levels. Red and blue in heatmap indicate relatively high and low values. Pearson’s correlation rates are indicated per protein and gene expression comparison and a cutoff at 0.5 is indicated as significant. (E) Western blot for TGFBI after treatment of U-343 MG cells with 2 different TGFBI shRNAs and selection of clones. (F) Relative cell numbers of U-343 MGa 31L cultured in conditioned media from U-343 MG and U-343 MG-shTGFBI cell cultures. (G) Relative cell amount of U-343 MGa 31L detected by MTT assay after treatment with recombinant TGFBI for 7 days.

To investigate intercellular communication mechanisms between U-343 cell lines through secreted proteins, we performed a set of combinatorial conditioned media experiments (Figure 5A and B). Conditioned media from U-343 MGa and U-343 MGa 31L promoted the proliferation of U-343 MG cells by 11% and 7%, respectively, while media from U-343 MGa Cl2:6 inhibited U-343 MG’s growth by 5% (Figure 5A). Conditioned media from U-343 MG increased the growth of U-343 MGa by 18%, but suppressed U-343 MGa 31L by 7%. No major effects via conditioned media were observed between the U-343 MGa cell lines except a suppressive effect by U-343 MGa Cl2:6 on U-343 MGa 31L by 9% (Figure 5B). Thus, we conclude that contact independent intercellular communication may also occur via secreted factors.

### Differential Levels of Secreted Proteins Detected by Mass Spectrometry and Confirmed by RNA-seq Analyses

Due to the identified effects via secreted factors we sought to identify proteins secreted from the U-343 cell lines by a secretome protein enrichment with click sugars (SPECS) analysis.^21^ U-343 MGa and U-343 MGa Cl2:6 had the most similar signatures of secreted proteins (*R*^2^ = 0.60 Pearson’s correlation), followed by U-343 MGa Cl2:6 and U-343 MGa 31L (*R*^2^ = 0.43), and U-343 MGa and U-343 MGa 31L (*R*^*2*^ = 0.25). U-343 MG displayed a noncorrelated secretome signature when compared to the U-343 MGa cell lines, where U-343 MGa Cl2:6 only had an *R*^*2*^ of 0.13, closely followed by U-343 MGa and U-343 MGa 31L with *R*^*2*^ values of 0.13 (Figure 5C; Supplementary Table S7).

To compare protein and gene expression levels, we analyzed the concordance between the secretome and RNA-seq data for the U-343 cell lines (Figure 5D). Four groups were designated based on Pearson’s correlation test between the protein and corresponding gene expression levels. For the first group, where both protein and gene expression levels were higher in U-343 MG, we display 16 secreted proteins (Figure 5D). For the third group, where both protein and gene expression levels were relatively higher in U-343 MGa cultures, we display 18 secreted proteins (Figure 5D). A complete list can be found in Supplementary Table S8.

To validate our findings and identify proteins from U-343 MG that could affect the growth of other U-343 MGa cultures, a functional approach was adopted for transforming growth factor beta-induced (TGFBI) and ADAM metallopeptidase with thrombospondin type 1 motif, 1 (ADAMTS1). We suppressed *TGFBI* in U-343 MG by 2 different short hairpin ribonucleic acids (shRNAs), named #1 and #2, and assessed the effect of conditioned media on the growth of U-343 MGa cultures. Western blot analysis confirmed TGFBI suppression by 2 shRNAs (Figure 5E). Conditioned media from U-343 MG with *TGFBI* suppression (shTGFBI #1 and #2) increased the growth of U-343 MGa 31L to 1.18 and 1.21 as compared to control, respectively (Figure 5F). Furthermore, conditioned media from shTFGBI #2 also increased the growth of U-343 MGa (Supplementary Figure S5A). Finally, the addition of recombinant TGFBI protein inhibited the growth of U-343 MGa cultures, especially U-343 MGa 31L (Figure 5G; Supplementary Figure S5B). Since U-343 MGa 31L acted as a temozolomide-resistant clone in coculture (Figure 1E), we tested if *TGFBI* suppression in U-343 MG affected this response. No significant difference was observed for the ratio of U-343 MGa 31L in cocultures with the 3 other U-343 lines including*TGFBI* suppressed or wildtype U-343 MG, or temozolomide treatment (Supplementary Figure S5C and D). Suppression of *ADAMTS1* in U-343 MG cells by shRNA and test of conditioned media decreased the growth of U-343 MGa 31L to 0.8 as compared to control, indicating that secreted ADAMTS1 has a proliferation promoting effect on U-343 MGa 31L (Supplementary Figure S5E and F).

Taken together, these findings demonstrate that the secretome signature may differ between cells in tumors with intratumoral heterogeneity that are involved in cell-to-cell communication. TGFBI and ADAMTS1 are 2 of these secreted proteins that affect cell proliferation in a coculture setting.

## Discussion

Intratumoral heterogeneity in glioblastoma is an issue that has been studied for a long time and has recently been fueled by novel findings based on single-cell omics.^2^ Several mechanistical studies have recently also highlighted consequences of intratumoral heterogeneity in glioblastoma, where for example subpopulations of neoplastic cells, based on *EGFR* mutation status, within the same tumor were shown to promote the growth by IL6 signaling.^14^ In a tumor evolutionary perspective, Ozawa et al.^25^ have placed glioblastoma gene expression-based subtypes within a framework where a proneural-like precursor is suggested to evolve and give rise to the other glioblastoma subgroups. In connection to this, different expression-based subsets, including stem like or mesenchymal, have been connected to tumor formation capacity related phenotypes.^26–28^ Furthermore, response to treatment has been connected with glioblastoma subtype transition.^29^ Together, this highlights the complexity and importance of a deeper understanding of intratumoral heterogeneity.

Here, we used the U-343 model, where the cell lines have been derived from a single glioblastoma, to identify how heterogeneous clones may evolve and phenotypically differ, and how interclonal signaling may exert co-evolutionary consequences. Our results from the U-343 model show that a common confounding clone may have existed that gave rise to phenotypically and genotypically divergent cells. Although it is not unlikely that a field of interacting differentially mutated cells would have been in place already at the initial stage of tumor development.^30,31^ In future studies, it will be of importance to investigate if the observed phenotypic differences between the cell lines have a genetic underlying cause since such has recently been described,^32^ and loss of *NF1* has been shown to play a role as a driver of mesenchymal subtype transition.^25^ But it should also be noted that cells have been described to appear in a spectrum of subtype states, which suggests a more variable situation than simple binomial states.^29^

The heterogeneity caused by tumor evolutionary divergence further raises the question of a need for combinatorial therapy approaches to target different populations. Theoretical models have shown that combinatorial approaches yield better results in diminishing the occurrence of drug resistance.^10^ Reinartz et al.^33^ have reported that the subclones from individual tumors exhibited heterogeneity in drug resistance. As we have shown here, U-343 cell lines differ in temozolomide sensitivity. The resistant clone, U-343 MGa 31L, expanded and became predominant after treatment, which is in line with other studies where preexisting clonal temozolomide insensitive populations have been shown to occur.^8,33^

Furthermore, communication between the U-343 cells also occurred via direct cell-to-cell contacts. We demonstrate that the mesenchymal-like cell line U-343 MG stimulates the growth of the non-mesenchymal-like U-343 MGa Cl2:6 cells, and in addition makes them more mesenchymal like with regard to expression of *FN1* and *SNAI2*. This demonstrates that a phenotype transition effect may exist between subsets of cells in a setting of intratumoral heterogeneity.

Here we studied the NOTCH signaling pathway since it has been shown to be involved in glioblastoma initiation.^34^ In the coculture experiments, we observed an increased cell proliferation of U-343 MGa Cl2:6 when cocultured with U-343 MG. Ablation of *NOTCH1* by CRISPR/Cas9 or shRNA in U-343MGa Cl2:6 lead to increased proliferation of U-343 MGa Cl2:6 in the coculture setting. This suggests that there is an underlying stimulatory effect sent from U-343 MG to U-343 MGa Cl2:6 cells, which is overruled by NOTCH1. But upon removal of the NOTCH1 protection, this effect becomes unleashed. Hence, this growth-promoting function by the U-343 MG cells needs to be further investigated.

Intratumoral cell communication has been shown to be important for overall tumor growth by interclonal stimulation.^14^ In this study, we demonstrated that the different U-343 cell lines indeed affected the proliferation of each other via secreted factors. We used a secretome protein enrichment with click sugars (SPECS), a method developed for secretome analysis, to identify secreted proteins by mass spectrometry without the interference of serum proteins in cell culture.^21,35^ In comparison with RNA-seq data, we found a high correlation between identified secreted proteins and the corresponding transcript levels. Candidate proteins that may contribute to the inhibitory effect of U-343 MG on U-343 MGa 31L cells were identified, and TGFBI and ADAMTS1 were selected for testing as potential effectors. In extension, the SPECS results also offer opportunities for studies of further secreted proteins. TGFBI has previously been shown highly expressed in mesenchymal subtype glioblastoma and associated with poor prognosis.^36^ Few reports are available on ADAMTS1 in glioblastoma, but in other cancer types it has been shown to affect several cancer phenotypes, including proliferation.^37^ Of note, it is also of importance to investigate the effects of the different secretomes on neighboring non-neoplastic cells, which has not been addressed in this work. For example, secreted factors from glioma cells that have transitioned to a mesenchymal subtype by loss of *NF1* have increased capacity to attract microglia.^4^

In summary, our study highlights the importance of intratumoral heterogeneity in glioblastoma. We show that genetically distinct tumor cell populations from a single glioblastoma can differ in drug sensitivity and with regard to astrocytic versus mesenchymal features and cell proliferation rates. Furthermore, these heterogeneous cells communicate via both direct cell-to-cell contacts and secreted proteins. An additional analysis of membrane-attached proteins and secreted factors may provide further insights into how glioblastoma cells can intercommunicate and if such factors constitute novel therapeutic targets.

## Supplementary Material

vdaa061_suppl_Supplementary_Tables

vdaa061_suppl_Supplementary_Material_and_Methods

vdaa061_suppl_Supplementary_Figure_S1

vdaa061_suppl_Supplementary_Figure_S2

vdaa061_suppl_Supplementary_Figure_S3

vdaa061_suppl_Supplementary_Figure_S4

vdaa061_suppl_Supplementary_Figure_S5

## Notes

**Authorship Statement.** Conception and design: M.G., S.H., M.N., and D.H.; development of methodology: M.G. and M.v.V.; analysis and interpretation of data (eg, statistical analysis, biostatistics, and computational analysis): M.G., M.N., and D.H.; writing, review, and/or revision of the manuscript: all authors; administrative, technical, or material support (ie, reporting or organizing data, constructing databases): M.G., M.v.V., J.Z., H.C., and S.H.; study supervision: M.N. and D.H..
