# Supplementary material for: Identification of functionally distinct and interacting cancer cell subpopulations from glioblastoma with intratumoral genetic heterogeneity
Source: Neurooncol Adv. 2020 May 27;2(1):vdaa061. doi: 10.1093/noajnl/vdaa061 (PMC7309246; doi:10.1093/noajnl/vdaa061)
Supplement: vdaa061_suppl_Supplementary_Material_and_Methods [file vdaa061_suppl_supplementary_material_and_methods.docx]

# Title: Identification of functionally distinct and interacting cancer cell subpopulations from a glioblastoma with intratumoral genetic heterogeneity

**Author list**: Min Guo^1*^, Marjolein van Vliet^1^, Jian Zhao^1^, Teresita Díaz de Ståhl^1^, Mikael S. Lindström^2^, Huaitao Cheng^1^, Susanne Heller^3,4^, Monica Nistér^1#^, Daniel Hägerstrand^1#^

**Author affiliations**: ^1^Department of Oncology-Pathology, Karolinska Institutet, SE-171 64 Solna, Sweden. ^2^Department of Medical Biochemistry and Biophysics, Karolinska Institutet, SE-171 21 Solna, Sweden. ^3^Department of Genetics and Pathology, Uppsala University, Rudbeck Laboratory, SE-751 85 Uppsala, Sweden. ^4^Uppsala Clinical Research Center (UCR), Uppsala University, Uppsala University Hospital, SE-751 85 Uppsala, Sweden

**Running title:** Intratumoral communication in glioblastoma

^#^These authors share senior responsibility

**Corresponding author:**

*Min Guo (email: [min.guo@ki.se](mailto:min.guo@ki.se), phone: +46 76 075 35 10)

Mailing address: Department of Oncology-Pathology, Karolinska Institutet, BioClinicum J5:30, Visionsgatan 4, Karolinska University Hospital, SE-171 64 Solna, Sweden

## Supplemental Material and Methods

### Cell culture conditions

The U-343 cell panel was previously derived as described from a glioblastoma that was divided into two parts, from which U-343 MG and U-343 MGa cells were initially established. The U-343 MG cells were established on plastic, while U-343 MGa was established on a feeder cell layer. U-343 MGa 31L and U-343 MGa Cl2:6 were later sub-cloned from U-343 MGa. The U-343 MG cells express FN1 and conversely the U-343 MGa cultures express GFAP. The opposed GFAP and FN1 gene expression patterns in glioblastoma cell lines were recently illustrated by Goudarzi et al., who also confirmed by STR profiling the single patient origin of the U-343 cells.^1^ Cells were maintained in Minimum Essential Medium (MEM) with 10% FBS and penicillin-streptomycin at 37°C with 5% CO_2_ and 5% O_2_ and over 95% humidity.

### RNA-seq analysis

RNA from the U-343 cells was extracted by PureLink^TM^ RNA Mini kit (Invitrogen^TM^). RNA-seq analysis was conducted at BEA (Bioinformatics and Expression Analysis) core facility, Novum, Karolinska Institutet, Sweden. To identify differentially expressed genes in each cell line, genes with values less than 0.5 in four cell lines were removed, then comparisons were performed between each line with the average of other three cell lines. Venn diagrams were generated using GeneVenn (<http://genevenn.sourceforge.net)>. Gene expression heatmaps were generated with the Heatmap module in GenePattern.^2^ CCLE data from 45 glioblastoma samples was downloaded from [www.broadinstitute.org/ccle](http://www.broadinstitute.org/ccle). Genes in CCLE cell lines were arranged according to correlation with *FN1* and *GFAP*, respectively, and the top 100 genes were used to create a heatmap and for further comparison with U-343 cell lines. Glioblastoma cell lines in *FN1*^high^*GFAP*^low^ and *GFAP*^high^*FN1*^low^ groups were arranged according to *FN1* expression (from high to low), subsequently according to GFAP expression (from low to high) in *FN1*^high /low^ subgroups. Single cell RNA-sequencing data of glioblastoma MGH28 and MGH29 was derived from Patel et al.^3^ Genes correlated with *FN1* or *GFAP* (correlation rate >0.2) in both MGH28 and MGH29 were selected and used to create a heatmap and for further comparison with U-343 cell lines. Single cells in *FN1*^high^*GFAP*^low^ and *GFAP*^high^*FN1*^low^ groups were arranged according to *FN1* expression (from high to low), subsequently according to GFAP expression (from low to high) in *FN1* ^high /low^ subgroups. U-343 genes were also compared with subtype-defining centroid genes as previously described.^1,4^ Each subtype is defined by a set of 210 centroid genes where a support index value of 100 means all the genes are altered in the positive direction, 0 means no change, and -100 means a full change in the negative direction.

### Genetic analysis

DNA was purified from the U-343 cells with GenElute Mammalian Genomic DNA Miniprep Kit (Sigma-Aldrich, MO, USA). For somatic copy number analysis, the DNA was hybridized to a SurePrint G3 Human CGH Microarray (Agilent Technologies Inc. CA, USA) using normal male genomic DNA as a reference. After hybridization the slide was scanned in a SureScan Microarray Scanner and Features were extracted with Agilent Feature Extraction Software Kit. Copy number alterations were analyzed with Agilent Genomic Workbench 7.0.4.0 (Agilent Technologies Inc. CA, USA).

### Generation of GFP labeled cells

EGFP-expressing lentivirus was acquired from Cyagen (Cyagen Bioscience Inc, CA, US) with a CMV promoter and Neomycin selection marker. After virus infection, EGFP (enhanced-GFP) expressing cells, from here on referred to as GFP labeled, were selected during 1 week by addition of G418 (500μg/ml, Sigma-Aldrich, MO, USA). Expression of GFP in cells was confirmed by using fluorescence microscopy and FACS.

### Lentiviral shRNA transfection

Lentiviral vectors with TGFBI targeting shRNA (TRCN0000062174, TRCN0000062175), ADAMTS1 targeting shRNA (TRCN00000286863), NOTCH1 targeting shRNA (TRCN0000003362), or shRNA control (shLacZ1650) were purchased from Sigma. Virus was generated as previously described.^5^ 1μg/ml puromycin was used for selection. Knockdown efficiency was determined one week after completed selection by Western blot.

### Generation of NOTCH1 knock out U-343 MGa Cl2:6 cells

Knock-out of *NOTCH1* in U-343 MGa Cl2:6 was performed by CRISPR/Cas9 based gene editing.^6^ The *NOTCH1* guide RNA (GGTGAGACCTGCCTGAATGGCGG) was designed with CHOPCHOP ([https://chopchop.rc.fas.harvard.edu/index.php](https://chopchop.rc.fas.harvard.edu/index.php)), and cloned into the CRISPR/Cas9 vector (Addgene, #48139). The *NOTCH1* CRISPR/Cas9 nuclease vector was transfected into U-343 MGa Cl2:6 by electroporation followed by selection with 1μg/ml of puromycin. Single cells were picked and expanded. Knock-out efficiency was validated by Western blot and Sanger sequencing of the guide target region in *NOTCH1*.

### Invasion assay

50,000 cells/chamber were seeded in BD Matrigel (BD Bioscience, MA, USA) coated invasion chambers and incubated for 24 or 48 hours. Subsequently, the invaded cells were fixed for 20 minutes in ice-cold methanol and mounted by Daco mounting solution containing DAPI (Daco Scientific Limited Company, UK). Images of nuclei from invaded cells were taken at 20X magnification in a fluorescence microscope and counted by ImageJ.^7^

### Cell proliferation assay

Cell proliferation in the presence of TGFBI recombinant protein was assessed by MTT assay. 3,000 cells/well were seeded in 96-well plates, then TGFBI recombinant protein (0, 5, 10μg/ml) was added on the second day. After 7 days of culture, the cells were stained with 500μg/ml MTT and the absorbance was measured at 570nm and 630 nm.

### Temozolomide sensitivity assay

Temozolomide was dissolved in DMSO (Sigma-Aldrich, Merck, DE). To assess temozolomide sensitivity, 3,500 or 10,000 cells/well were seeded in 96-well plates treated with temozolomide (concentration range from 0 to 2000 μM). Changes in cell amount were assessed by MTT assay after treatment for 4 days or 8 days.

### Temozolomide treatment in a U-343 co-culture model

In the co-culture experiments, the four U-343 cell lines were cultured in 4 different combinations containing all cell lines, but only one cell line was GFP-labeled in each combination. Cells were seeded at equal amounts (5,000 cells for each cell line, yielding a total of 20,000 cells/well) in the presence or absence of 200 μM temozolomide. After 5 and 10 days the total number of cells was counted by hemocytometer and the GFP-cell ratio was determined by using FACS for each combination. The final number of GFP-labeled cells was extrapolated from the ratio of GFP-labeled cells and the total amount of cells.

### Conditioned media experiments

10 ml of conditioned media was collected from 500,000 cells in a 10 cm culture dish after 72 hours of incubation in MEM with 10% FBS. In experiments, conditioned media was added to U-343 cell lines as a 1:1 ratio with unconditioned media every second day to minimize nutrient depletion risk. Then cell number was calculated and compared after culturing for 7 days.

### Cell-to-cell co-culture experiments

Non GFP-labeled cells (200,000 cells/well for U-343 MG, 600,000 cells/well for U-343 MGa Cl2:6 in 6-well plate) were seeded on the bottom and incubated for 48h, followed by addition of GFP-labeled cells (40,000 cells/well) on top. After 5 days of co-culture cell number was counted by hemocytometer and the percentage of GFP-labeled cells measured by FACS. The final number of GFP-labeled cells was extrapolated from the ratio of GFP-labeled cells and the total amount of cells. To further compare the gene expression and protein changes, GFP-labeled cells were sorted and analyzed by qPCR and Western blot. To monitor morphology changes of GFP-labeled cells in co-culture, real-time photos were taken at different time point (10 hours and 36 hours) with Incucyte live-cell analysis system (PerkinElmer, USA).

### Secretome analysis by Click-iT

To detect secreted proteins the Click-iT Protein Enrichment Kit for click chemistry capture of azide-modified proteins was used (Life Technologies, NY, USA). To label newly synthesized proteins, cells were incubated with 10 ml of Methionine free DMEM supplemented with either Methionine or AHA (Sigma-Aldrich, MO, USA) for 48 hours. Media was subsequently concentrated to 100μl using Amicon Ultra-15 centrifugal filters (Millipore). The concentrated conditioned media was treated according to a previously described Click-It protocol,^8^ where AHA labeled proteins were bound to click-beads. Bound proteins were subsequently digested and identified by mass-spectrometry. Beads incubated with proteins from the unlabeled corresponding cell cultures were used as controls to be able to subtract background binding. The number of peptides mapping to each corresponding protein was used in further comparison with RNA-seq data.

### Comparison of secretome data and RNA-seq data

The secretome data from U-343 was derived according to a previously described Click-It protocol,^8^ and identified by mass-spectrometry. Proteins with peptides found in the negative control of all cell lines were removed from the list. Proteins remaining in the positive samples were kept and *z*-score was calculated. Proteins with a *z*-score in the U-343 MG higher than the average in the U-343 MGa cultures (cutoff=1) were regarded as “higher secreted proteins in U-343 MG”. The proteins with average *z*-score in the three U-343 MGa cultures higher than in U-343 MG (cutoff=1) were regarded as “higher secreted proteins in U-343 MGa cultures”. The gene expression *z*-score of these genes was also calculated according to the RNA-seq data. The correlation between protein and gene expression level was analyzed by Pearson’s correlation.

## References

**1.** Goudarzi KM, Espinoza JA, Guo M, et al. Reduced expression of PROX1 transitions glioblastoma cells into a mesenchymal gene expression subtype. *Cancer Res.* 2018; 78(20):5901-5916.

**2.** Reich M, Liefeld T, Gould J, Lerner J, Tamayo P, Mesirov JP. GenePattern 2.0. *Nat Genet.* 2006; 38(5):500-501.

**3.** Patel AP, Tirosh I, Trombetta JJ, et al. Single-cell RNA-seq highlights intratumoral heterogeneity in primary glioblastoma. *Science.* 2014; 344(6190):1396-1401.

**4.** Verhaak RG, Hoadley KA, Purdom E, et al. Integrated genomic analysis identifies clinically relevant subtypes of glioblastoma characterized by abnormalities in PDGFRA, IDH1, EGFR, and NF1. *Cancer Cell.* 2010; 17(1):98-110.

**5.** Hagerstrand D, Tong A, Schumacher SE, et al. Systematic interrogation of 3q26 identifies TLOC1 and SKIL as cancer drivers. *Cancer Discov.* 2013; 3(9):1044-1057.

**6.** Ran FA, Hsu PD, Wright J, Agarwala V, Scott DA, Zhang F. Genome engineering using the CRISPR-Cas9 system. *Nat Protoc.* 2013; 8(11):2281-2308.

**7.** Schneider CA, Rasband WS, Eliceiri KW. NIH Image to ImageJ: 25 years of image analysis. *Nature methods.* 2012; 9(7):671-675.

**8.** Eichelbaum K, Winter M, Berriel Diaz M, Herzig S, Krijgsveld J. Selective enrichment of newly synthesized proteins for quantitative secretome analysis. *Nat Biotechnol.* 2012; 30(10):984-990.

## Supplemental figure legends

### Figure S1. Temozolomide sensitivity in each U-343 cell line

### (A) Temozolomide sensitivity profiles of U343 cell lines measured by MTT assay. 3,500 cells were seeded in 96-well plates, and treated with temozolomide (concentration range from 0 to 2000 μM) for 8 days. (B) Same as A, but with 10,000 cells and treated for 4 days.

### Figure S2. The expression of EMT genes in each U-343 cell line by RNA-seq analysis

**(A-D)** Top 20 hallmark gene categories processed by GenePattern among the average of U-343 MG genes (A), U-343 MGa genes (B), U-343 MGa 31L genes (C) and U-343 MGa Cl2:6 genes (D). **(E)** Heatmap shows the expression of EMT genes derived from each U-343 cell line. **(F)** Overlap of EMT genes between the different U-343 cell lines. **(G)** U-343 highly expressed genes compared with subtype-defining centroid genes. A support index value of 100: all genes altered in positive direction; 0: no change; -100: all genes altered in negative direction.

### Figure S3. Exclusive focal alterations in each U-343 cell line by copy number analysis and associated gene expression data by RNA-seq

**(A)** Exclusive focal alterations in each U-343 cell line by copy number alteration. **(B)** Differentially expressed genes specifically changed in one of the four U-343 cell lines both at genomic level (left panel) and at RNA gene expression level (right panel). Relatively high/low levels are shown in red/blue in the heat map.

### Figure S4. Generation of NOTCH1 knockout U-343 MGa Cl2:6 cells by CRISPR/Cas9 gene editing

**(A)** The sequencing and the location of single guide RNAs (gRNA) in exon 2 of human NOTCH1 gene. **(B)** Sanger sequence alignments around the gRNA targeting sites in U-343 MGa Cl2:6 NOTCH1 Wild type and NOTCH1-KO#1 and NOTCH1-KO#3 cells. The sequencing was performed from both 5’- and 3’-ends. The green colored bases are mismatched sites.

### Figure S5. The effect of TGFBI on U-343 MGa cultures

**(A)** Relative cell number of U-343 MGa and U-343 MGa Cl2:6 cultured in conditioned media either from U-343 MG-shcontrol or from U-343 MG-shTGFBI#1 or #2 cell cultures. **(B)** Relative growth, measured by MTT assay of U-343 MG, U-343 MGa, U-343 MGa 31L, and U-343MGa Cl2:6 cells, treated for 4 days with 2.5 μg of recombinant TGBI or control. The growth of all 4 lines was significantly inhibited by TGFBI, although a stronger effect was seen for U-343 MGa 31L **(C** and **D)** The ratio of U-343 MGa 31L after coculture with a combination of U-343 MGa, U-343 MGa Cl2:6 and U-343 MG (shRNA control, shTGFBI#1 or #2) for 10 days with DMSO (C) or temozolomide treatment (D). **(E)** Westernblot showing decrease of ADAMTS1 protein caused by shADAMTS1 in U-343 MG. **(F)** Relative cell number of U-343 MGa 31L, U-343 MGa, and U-343 MGa Cl2:6 cultured in conditioned media either from U-343 MG-shcontrol or from U-343 MG-shADAMTS1 cell cultures. Significant inhibition was observed only of U-343 MGa 31L cells.
