# Supplementary figures and images for: Identification of functionally distinct and interacting cancer cell subpopulations from glioblastoma with intratumoral genetic heterogeneity
Source: Neurooncol Adv. 2020 May 27;2(1):vdaa061. doi: 10.1093/noajnl/vdaa061 (PMC7309246; doi:10.1093/noajnl/vdaa061)

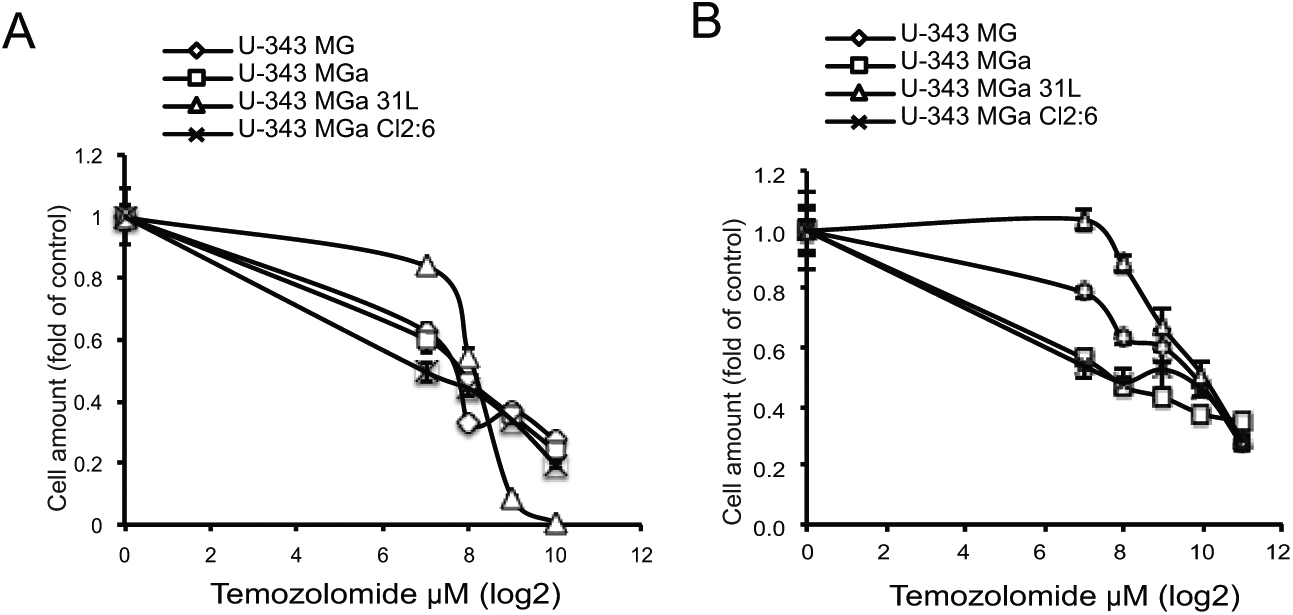

Supplement: vdaa061_suppl_Supplementary_Figure_S1 [file vdaa061_suppl_supplementary_figure_s1.jpeg]

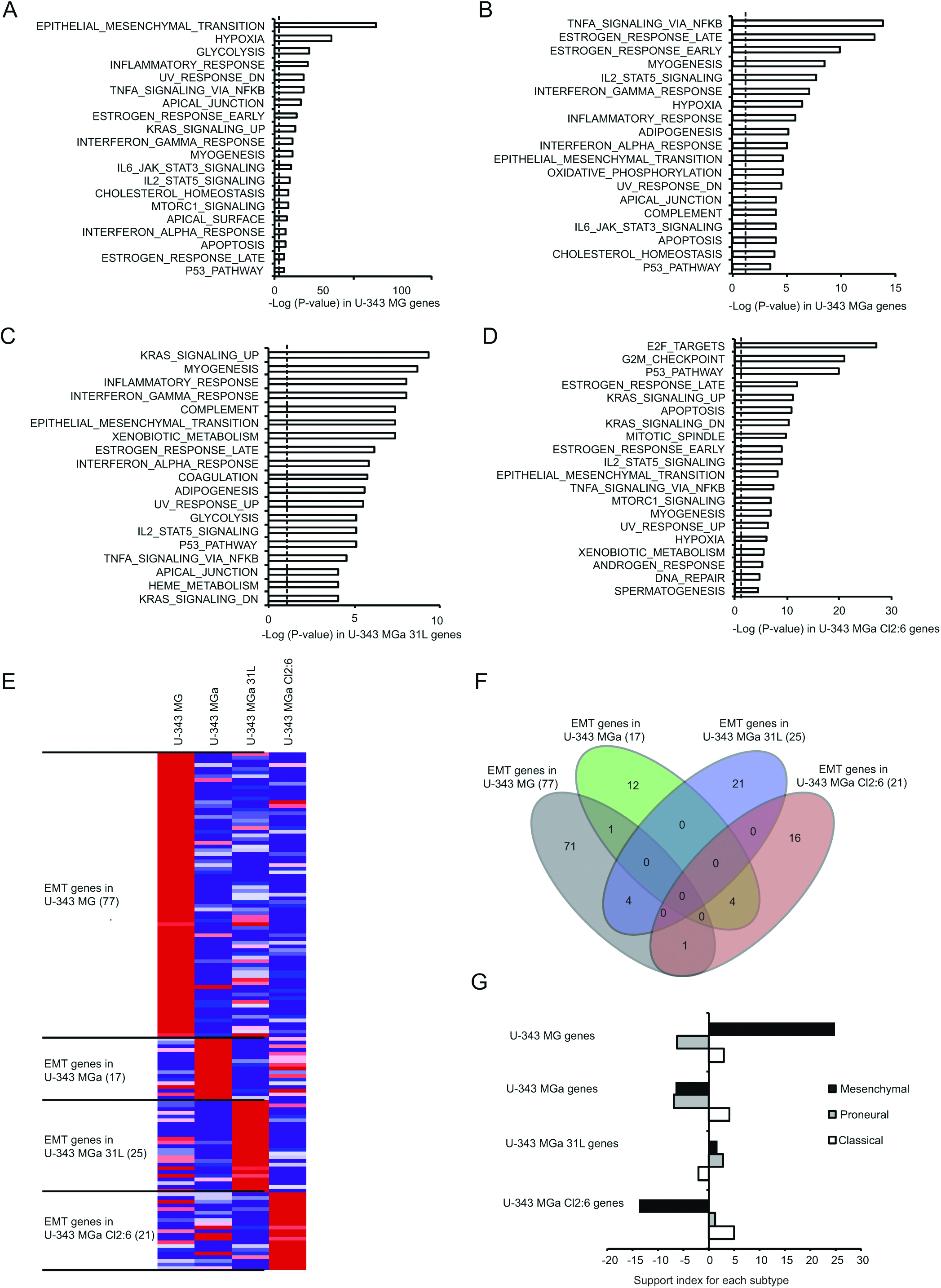

Supplement: vdaa061_suppl_Supplementary_Figure_S2 [file vdaa061_suppl_supplementary_figure_s2.jpeg]

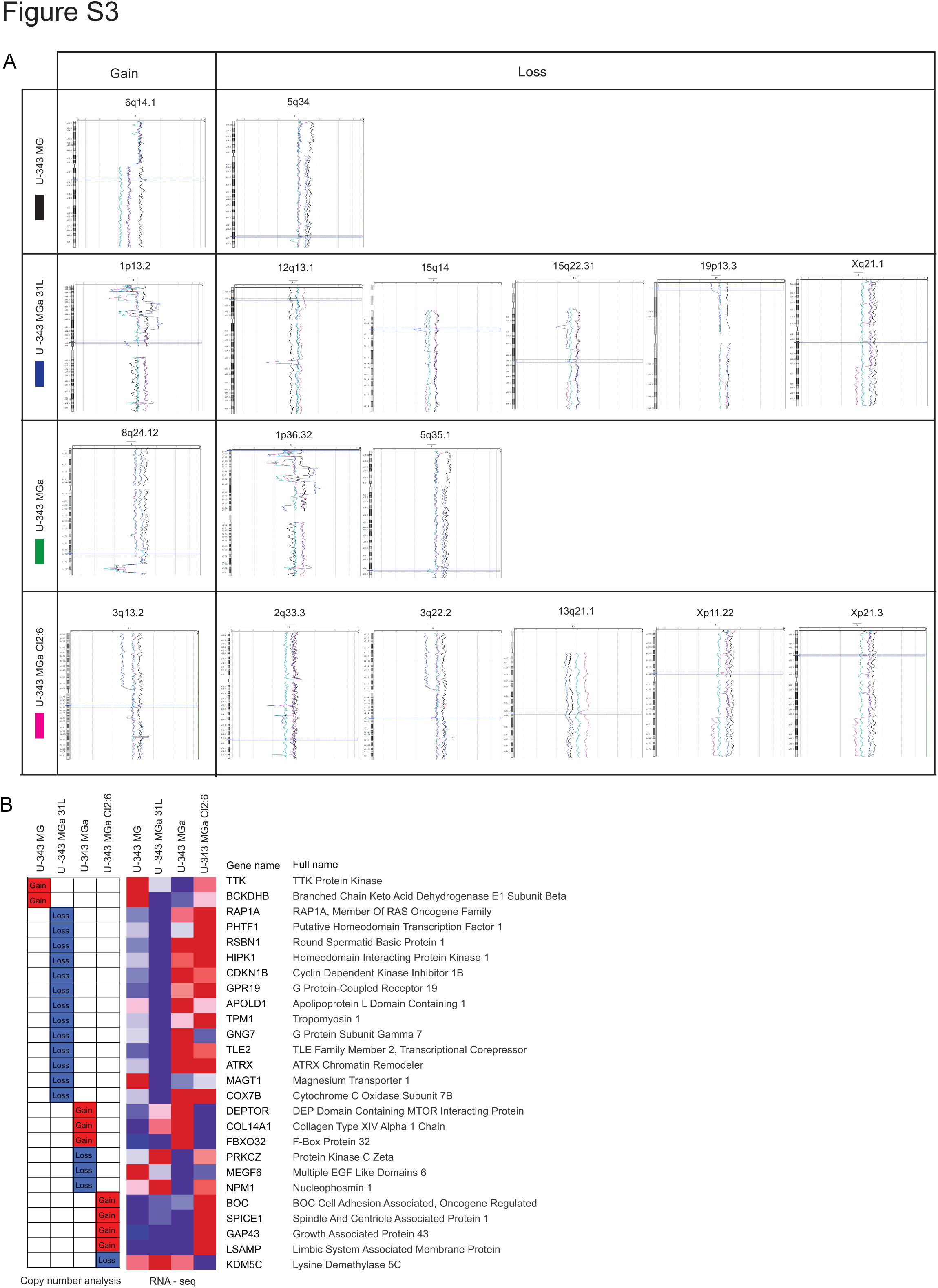

Supplement: vdaa061_suppl_Supplementary_Figure_S3 [file vdaa061_suppl_supplementary_figure_s3.jpeg]

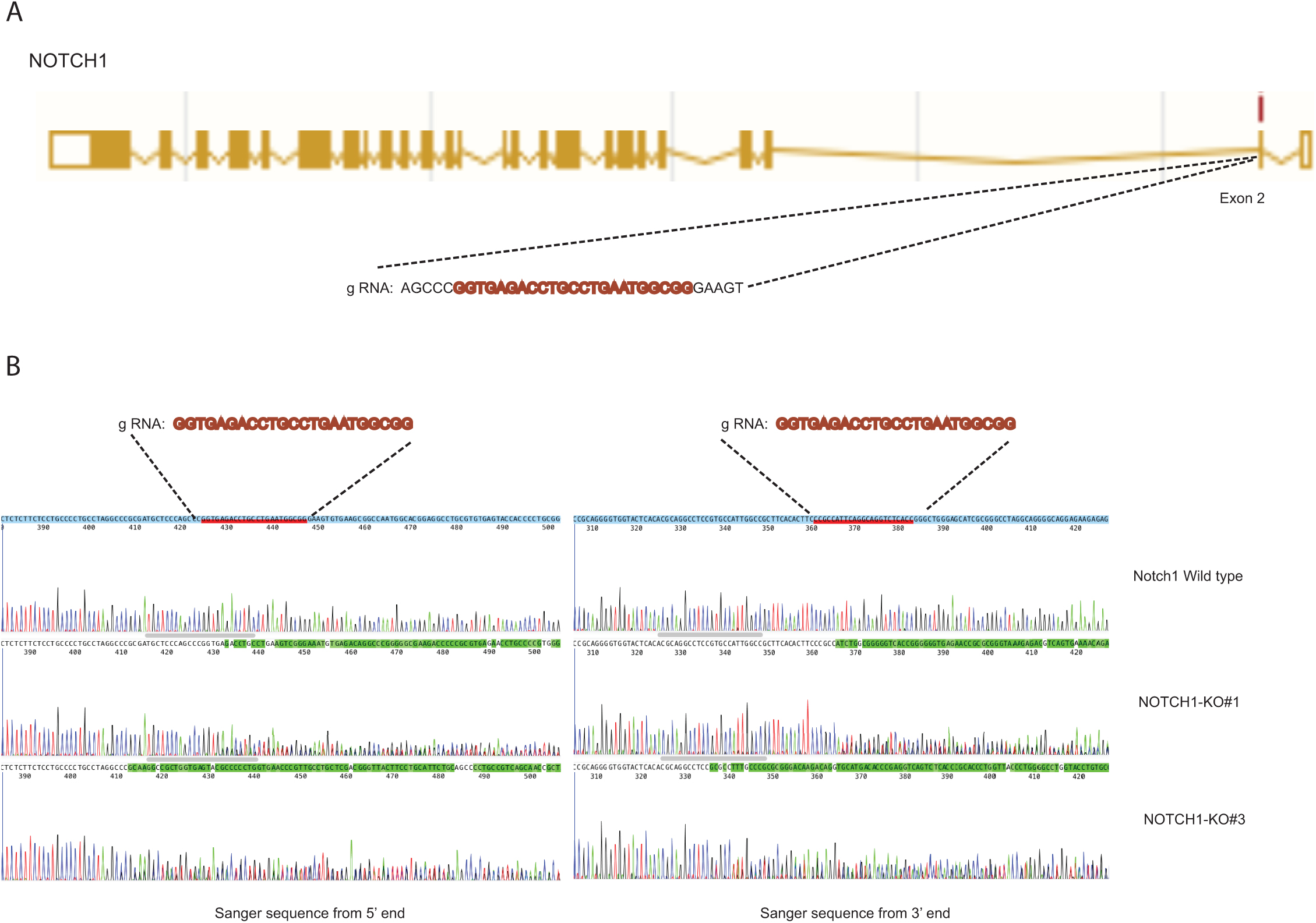

Supplement: vdaa061_suppl_Supplementary_Figure_S4 [file vdaa061_suppl_supplementary_figure_s4.jpeg]

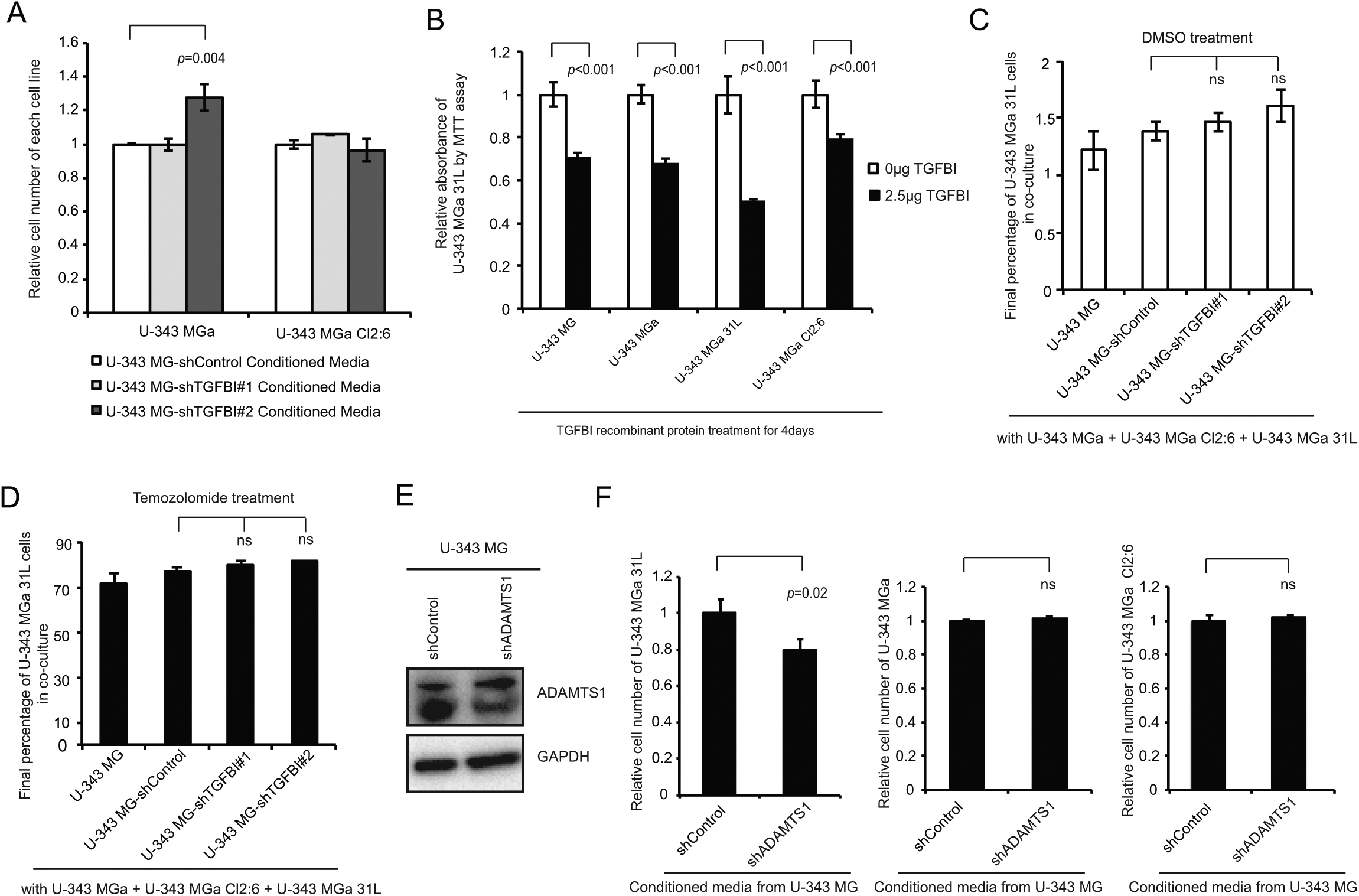

Supplement: vdaa061_suppl_Supplementary_Figure_S5 [file vdaa061_suppl_supplementary_figure_s5.jpeg]
